# Regulating addictive algorithms and designs: protecting older adults from digital exploitation beyond a youth-centric approach

**DOI:** 10.3389/fpsyg.2025.1579604

**Published:** 2025-07-28

**Authors:** Yu Yao

**Affiliations:** School of Law, China Jiliang University, Hangzhou, China

**Keywords:** digital addiction, addictive algorithms and designs, older adults, mental health, physical health, digital vulnerability, digital exploitation

## Abstract

As digital technologies evolve, addictive algorithms and designs (aADs) have become a significant public health concern, particularly for older adults, a demographic often overlooked in digital addiction (DA) discourse. Compared to younger users, some older adults may face increased susceptibility to aADs due to factors such as cognitive changes, social isolation, physical comorbidities, or limited digital literacy. These designs, which exploit features like infinite scrolling, are associated with higher risk of compulsive use and may correlate with long-term impacts on cognitive health, social functioning, and quality of life for certain subgroups of older adults. Existing regulatory frameworks predominantly focus on protecting minors, often neglecting the distinct needs and vulnerabilities within aging populations. This paper advances a pluralistic governance framework that recognizes the diversity among older adults. Recommended measures include behaviorally informed disclosures, algorithmic transparency, tailored digital literacy programs, culturally responsive safeguards, and antitrust actions against exploitative design. Age-inclusive regulation is urgently needed to address potential risks of digital dependency, mental health concerns, and social inequalities among aging populations. Addressing these challenges requires collaboration across sectors to ensure digital environments support autonomy and well-being at all ages.

## 1 Introduction

Digital technologies’ rapid development has intensified attention competition, prompting platforms to deploy addictive algorithms and designs (aADs) that maximize engagement (Bhargava and Velasquez, 2021). Evolved from persuasive technology, a field once intended to help users achieve beneficial goals without coercion or deception (Fogg, 1998), aADs are algorithm and design features that exploit psychological vulnerabilities to maximize engagement. This results in users spending time or committing attention beyond what is expected, convenient, or healthy for them (Agencia Española de Protección de Datos (AEPD), 2024), often prioritizing platform profits over user welfare and potentially fostering digital addiction (DA).

While regulatory and public health efforts on aADs primarily target children and adolescents (Costello et al., 2023), older adults are a neglected yet growing segment of digital users (Xiang and Lu, 2024). Contrary to stereotypes of technological resistance, older adults are increasingly engaged online (Vaportzis et al., 2017). In 2020, approximately 61% of Europeans aged 65–74 reported recent internet use (Eurostat, 2021), and by December 2024, 14.1% of China’s 1.1 billion Internet users were aged 60 or above (China Internet Network Information Center, 2025). A report by Xinjing Think Tank found over 43% of Chinese older adults spend more than 4 h online daily. Among them, more than 63% experienced occasional physical fatigue or vision problems, and 17.26% reported frequent symptoms (Xinjing Think Tank, 2024).

A Beijing-based study surveyed 329 adults aged 60 + actively using platforms such as WeChat, Tiktok, and Taobao (Sun, 2023). Using a validated Chinese WeChat Addiction Scale (Cronbach’s *α* = 0.908) (Montag et al., 2018), thresholds for high engagement and addiction were set using Zhao and Xue’s (2014) mean-standard deviation method. This method, assuming normal distribution, categorized users as normal (≤3.84), high engagement (3.84–4.55), or addicted (>4.55) based on a national sample (mean = 3.12, SD = 0.71), with high engagement at one SD above the mean and addiction at two SDs. Results showed 54.1% of participants had high engagement and 6.4% met addiction criteria, significantly higher than general national rates (15.96 and 2.13% respectively). Crucially, the study identified aADs like personalized recommendations and variable rewards as strong predictors of addiction, with older adults more affected than younger controls (*p* < 0.001). However, the national sample used for setting these thresholds may not fully reflect older adults’ distinct usage patterns, and self-reported data can introduce potential biases. Furthermore, while these findings from China offer important insights, their generalizability across diverse cultural and socio-political contexts remains a critical consideration.

While concern about aADs’ impact on older adults is growing as highlighted by such studies, it is essential to recognize their diversity. Differences in digital skills, cognitive resilience, and social backgrounds shape how individuals respond to aADs. Rather than treating all older adults as equally vulnerable, this paper examines specific conditions that increase susceptibility within subgroups. By identifying distinct risks in digital environments shaped by aADs, this paper examines the gaps in existing regulatory frameworks and outlines a set of responses aimed at preventing digital harm while preserving user autonomy.

## 2 Why certain older adults are particularly vulnerable

Not all older adults are equally at risk, but specific subgroups are especially susceptible to aADs (Rossi et al., 2024), increasing the likelihood of DA. While many adolescents are vulnerable due to neurobiological immaturity (Schepis et al., 2008; Kuss et al., 2013) and limited self-regulation (Howard et al., 2025; Burnell et al., 2023), older adults’ underexplored vulnerabilities (Bongard-Blanchy et al., 2021; Ener et al., 2020; Koh and Seah, 2023) warrant greater attention, especially given their diverse backgrounds and differential digital literacy.

### 2.1 How aADs manipulate the mind

aADs intensify engagement by exploiting cognitive and psychological processes. Key tactics leverage principles like operant conditioning (Skinner, 1938), employing variable ratio reinforcement schedules akin to gambling (Király et al., 2018; Griffiths and Nuyens, 2017). Unpredictable rewards, such as likes or new content, trigger dopamine-driven reward anticipation (Wise and Rompre, 1989), reinforcing habitual use (Graybiel, 2008). Features like infinite scroll and autoplay similarly to Brian Wansink’s “bottomless bowl” experiment (Wansink et al., 2005), where the absence of natural stopping cues encouraged unconscious overconsumption. By removing clear endpoints, these design elements diminish users’ ability to disengage, thereby extending screen time (Mdner et al., 2021).

Cognitive biases amplify compulsive engagement. The sunk cost fallacy (Arkes and Blumer, 1985) discourages users from quitting, while commitment bias (Back and Flache, 2008), reinforced by sign-in rewards or leveling systems, promotes continued use. The Zeigarnik effect (Zeigarnik, 1927) exploits incomplete tasks to prompt return visits, and the endowment effect (Kahneman et al., 1991) fosters attachment to digital assets like Snapchat streaks, which reinforces users’ platform reliance. For some older adults, particularly those experiencing age-related changes in cognitive flexibility, sludge tactics, such as hidden unsubscribe buttons (Thaler, 2018; Mills et al., 2023), can pose significant extra barriers. Personalized recommendation algorithms (PRAs) analyze user behavior to maximize reward anticipation, creating a dopamine-driven feedback loop.

Neurologically, excessive internet use is associated with reductions in gray matter in the ventral striatum (He et al., 2017; Montag et al., 2017), which may impair reward processing and contribute to DA (Westbrook et al., 2021). PRAs reinforce the cue-behavior-reward loop, driving habitual engagement. Experimental fMRI studies demonstrate that personalized content directly modulates brain activity in key regions, including sub-components of the default mode network (DMN) (Su et al., 2021), the ventromedial prefrontal cortex (vMPFC), dorsal anterior cingulate cortex (dACC), caudate nucleus, and thalamus. This modulation is associated with reduced conscious awareness (Cavanna and Trimble, 2006) and cognitive control (Carter and van Veen, 2007). It is hypothesized that such disruption may also weaken neural coupling between the DMN and areas like the anterior cingulate cortex (ACC) and precuneus, inducing an immersive, passive consumption state that facilitates addictive usage patterns.

### 2.2 Cognitive decline

Some older adults, particularly those who experience age-related changes in attention, memory (Abdulrab and Heun, 2008; Tandetnik et al., 2015), and executive functions (Hu et al., 2017; Viviano and Damoiseaux, 2020), may find it harder to recognize aADs, manage impulses, or disengage from engaging content (Xu et al., 2023; Khang et al., 2013). Unlike younger cohorts raised in digital environments, many older adults developed their cognitive frameworks in a pre-digital era (Johnson, 2022), potentially limiting their ability to recognize and resist habit-forming aADs patterns. Empirical studies support this. Xiao and Wang (2024) found that older adults identified aADs less accurately than those aged 18–25, despite both groups expressed strong negative attitudes. Another study showed individuals under 40 with at least a high school education better understood aADs (Bongard-Blanchy et al., 2021). This intergenerational awareness gap can contribute to increased susceptibility for certain older users in algorithm-driven environments.

### 2.3 Psychological vulnerabilities and physiological comorbidities

Loneliness and social isolation, prevalent among some older adults, are significant risk factors for problematic digital engagement (Meshi et al., 2020). aADs can exploit these vulnerabilities, potentially deepening isolation (Kraut et al., 1998). Problematic internet use is closely associated with depression, anxiety, and suicidal thoughts in older adults (Chen et al., 2024; Kuss and Griffiths, 2011; Cunningham et al., 2021). A Turkish study found a significant positive correlation between DA and increased depression and anxiety among older adults (Karaş et al., 2023). While individuals experiencing depression may turn to digital tools for symptoms relief, aADs-driven overuse can negatively impact their daily lives and social interactions, potentially creating a vicious cycle (Ko et al., 2012; M’hiri et al., 2015).

For some older adults, physical comorbidities (e.g., cardiovascular disease, arthritis, diabetes) that can lead to limited mobility and increased sedentary behavior (Kraut et al., 1998; Sun and Zhou, 2021) are associated with heightened digital engagement and aADs exposure. Sleep disorders, also prevalent alongside aging-related circadian disruptions for some individuals (Stepnowsky and Ancoli-Israel, 2008), can be influenced by prolonged screen use. Research indicates a negative correlation between DA and sleep quality among older smartphone users (Jia et al., 2023). Screen light exposure inhibits melatonin secretion (Nagare et al., 2019; Höhn et al., 2024), potentially promoting circadian misalignment (Chellappa et al., 2011) and worsening health issues (Šmotek et al., 2020). It’s crucial to acknowledge these relationships are often bidirectional, with overall lifestyle and pre-existing medical conditions also playing significant roles.

### 2.4 Long-term effects

Sustained problematic digital engagement poses serious long-term risks, particularly for cognitive and social health. A 13-year, large-scale longitudinal study (Yuan et al., 2023) of 462,524 UK middle-aged and older adults associated excessive daily screen time with increased dementia risk and hippocampal volume reduction. In China, a study of 303 adults over 55 found that DA correlated with reduced communication abilities and diminished social functioning through increased loneliness and diminished hope (Jia et al., 2022). Beyond individual harm, DA increasingly affects family dynamics. Jia’s research (Jia et al., 2023), using a “feedback model” of intergenerational relations, found that older adults, especially migrant grandparents, and primary caregivers, who provide support but receive little emotional reciprocity, were more prone to problematic digital use. In these cases, platforms often serve as emotional substitutes, deepening dependency and social withdrawal.

These patterns are connected to a reduced quality of life, marked by increased anxiety, lower life satisfaction, and disrupted daily routines. In a qualitative study conducted in Changsha, China, involving 20 older participants aged 60+ (Lu and Zhou, 2025), family members described older relatives spent entire afternoons watching algorithmically recommended videos, gradually withdrawing from social interaction and household life. Such trends highlight the urgent need for regulatory interventions targeting aADs to address both personal and relational dimensions of digital harm.

## 3 Current regulatory landscape and its gaps

### 3.1 A youth-centric regulatory paradigm

Despite the risks aADs pose, existing regulatory measures primarily target minors. In the United States, legislative efforts like New York State Senate (2023) aim to prevent social media platforms from offering algorithmically curated content to users under 18 without parental consent. While Europe’s proposed Digital Fairness Act seeks to address problematic online practices for all consumers, including addictive design and data-driven vulnerability, its legislative focus and stakeholder narratives have so far centered on youth protection. Asian jurisdictions have also adopted youth-specific strategies. South Korea’s now-repealed “Shutdown Law” (2011–2021) prohibited late-night gaming for children under 16 (Choi et al., 2018), while Japan’s Kagawa Prefecture set screen time limits for minors. China has adopted a relatively broader approach. Its revised Law on the Protection of Minors (Standing Committee of the National People’s Congress of China, 2020) prohibits platforms from providing addictive content to minors (Article 74), and the Regulations on the Protection of Minors Online (State Council of China, 2023) mandate anti-addiction features in platform design. Significantly, China’s 2022 Provisions on the Management of Algorithmic Recommendations in Internet Information Services (Cyberspace Administration of China et al., 2021) established a general duty for providers to avoid creating addictive systems or inducing excessive user engagement across all age groups. However, its practical implementation and enforcement remain disproportionately focused on minors. This selective regulatory approach treats aADs as a youth-specific hazard rather than a universal, cross-generational challenge.

### 3.2 Why existing regulations are insufficient

Despite some progress in aADs governance, current regulatory strategies have conceptual, functional, and cultural limitations that undermine their applicability to older adults.

First, design-specific prohibition remains the dominant regulatory approach. Measures like the U.S. Social Media Addiction Reduction Technology (SMART) Act (U.S. Senate, 2019) propose banning features such as infinite scroll. While such features intensify compulsive use, their deep integration into digital habits makes blanket prohibitions hard to enforce and potentially infringes on user autonomy. Unlike minors, older adults are legally classified as fully capable consumers, necessitating regulations that balance protection with respect for autonomy, responsive to varied vulnerabilities among older adults.

Second, the broader socio-economic costs of DA are often underestimated. Excessive technology use is linked to mental health issues, leading to both direct costs, such as healthcare expenses, and indirect economic losses, including decreased productivity and heightened risks of substance abuse (Day, 2022). Yet current regulatory models often treat aADs as isolated design flaws rather than structural market failures. A more integrated framework, combining consumer protection, antitrust tools, behavioral design accountability, and digital literacy promotion, is needed to address the full ecosystem of harms.

Third, current governance frameworks are culturally and socially limited. Western regulations often emphasize consent, privacy, and individual responsibility, while many East Asian systems prefer more paternalistic, public-interest approaches. Neither fully addresses the emotional, familial, and community-based factors associated with DA among older populations, such as loneliness, intergenerational disappointment, or rural digital marginalization. Banning addictive design features may be effective for minors in formal institutions, but far less so for older adults who rely on digital engagement as a substitute for social interaction. A one-size-fits-all regulatory model lacks cultural sensitivity and fails to adapt to diverse user needs.

To bridge this gap, we must move beyond youth-centric models, promoting an age-inclusive, cross-cultural governance paradigm.

## 4 Discussion

### 4.1 Regulating aADs for older adults: balancing protection and autonomy

The debate on regulation of aADs among adults is contentious, with some arguing for individual responsibility in digital consumption. However, this view overlooks fundamental ethical concerns regarding aADs. Autonomy, philosophically, is the capacity to voluntarily govern one’s life based on reasons and intentions, beyond mere stimulus–response behavior or immediate impulses (Vugts et al., 2020; Friedrich et al., 2018). aADs erode this by using predictive analytics and reinforcement loops to subtly shape behavior, diminishing self-directed decision-making. Presuming adults can always regulate their digital habits ignores how aADs impair rational agency. Similar to how contract law invalidates coerced agreements, regulatory frameworks must recognize that aADs-influenced digital behaviors may not reflect genuine autonomy. The state has a duty to intervene when external manipulation harms individual autonomy (Singer, 1995).

Proportionate oversight of aADs, similar to gambling regulation, is warranted to prevent large-scale behavioral manipulation. For adults, this aims to ensure informed, deliberate choices in transparent digital spaces, not to restrict access. Regulatory strategies should protect vulnerable groups while respecting individual autonomy, especially in culturally diverse societies. While autonomy is a universal principle, its practical expression can be shaped by cultural norms and family-based interdependence, which should inform regulatory design. Given significant potential harm despite limited direct causal evidence, regulatory interventions should follow the precautionary principle (Guida, 2021), advocating for prudent and proportionate measures that balance public welfare and industry innovation.

### 4.2 Potential regulatory interventions

#### 4.2.1 Behaviorally informed risk disclosure

Traditional disclosures like lengthy terms of service are ineffective, especially for older users with reduced cognitive flexibility. Vague or overly detailed notices often cause cognitive overload (Ben-Shahar and Schneider, 2011). Drawing from established evidence on the efficacy of simplified consumer information, such as the model of nutrition labels (Feunekes et al., 2008) and emerging research on privacy labels (Kelley et al., 2010), regulators could mandate concise, accessible “aADs Labels” in app stores or onboarding interfaces. These should feature an addiction risk score (e.g., a color-coded 1–5 scale) assessed by independent bodies, a time commitment indicator showing typical usage, and clearly summarized disengagement tools (e.g., time limits, notification controls). While the direct efficacy of aADs labels for older adults requires further empirical validation, their design is grounded in principles proven effective in promoting informed decision-making across various consumer contexts.

#### 4.2.2 Algorithmic transparency and oversight

Algorithmic transparency is essential for user protection. A national survey in China with 6,941 respondents found that 54.28% preferred simple algorithmic explanations, 38.49% wanted more detail, and 76.48% favored human review for problematic outcomes (Xu and Cheng, 2022). This data supports a tiered explanation model where platforms first outline algorithmic purpose and scope in non-technical terms, then provide summarized descriptions of personalization mechanisms. For disputes, full interpretability, including data sources, parameter relevance, and human-led review options, is crucial. To ensure accountability, independent audits and algorithmic impact assessments should be required for high-risk systems, especially those targeting users’ emotional or cognitive vulnerabilities.

Achieving transparency demands a careful balance with user privacy. Platforms should adopt privacy-preserving techniques such as differential privacy and ensure users have clear, accessible settings to manage data sharing preferences. While transparency measures entail substantial costs, platforms should bear these as compliance responsibilities. Implementing these recommendations faces real-world trade-offs, including potential platform resistance due to costs and competitive concerns. A pragmatic phased approach, beginning with high-risk aADs, alongside clear regulatory guidelines and practical incentives, is essential to ensure transparency leads to meaningful accountability.

#### 4.2.3 Digital literacy with a focus on aADs

Effectively responding to aADs requires not only technical skills but also critical digital literacy, which is the ability to recognize and navigate manipulative design features like variable rewards, and emotional targeting. Older adults, a highly diverse demographic, engage with digital platforms under varied cognitive, social, and cultural conditions. Public initiatives should promote inclusive literacy programs that enhance users’ critical awareness across all life stages. Rather than assuming a universal deficit, these efforts should build on existing strengths and experiences. Support can be strengthened through intergenerational learning, peer exchanges within communities, and integrating digital education into social services. Policymakers and industry actors should also foster the design of inclusive, transparent digital environments that respect diverse needs and promote autonomy for all users.

#### 4.2.4 Culturally responsive regulation

Effective regulation must reflect cultural and social contexts. In many East Asian societies, older adults’ platform interactions are shaped by emotional expectations, caregiving responsibilities, or intergenerational communication patterns. These behaviors do not indicate inherent vulnerability, but rather highlight a need for contextually appropriate and socially informed safeguards. To support meaningful engagement, platforms could offer optional tools that allow users to share selected settings or content explanations with trusted individuals. These tools must be governed by clear consent mechanisms and strong privacy protections. Such features can enhance users’ ability to understand and manage aADs without diminishing their independence or dignity. Importantly, all regulatory interventions should follow the principle of minimal intrusion and proportionality to demonstrated risks. They should aim to support rather than replace individual decision-making. Culturally responsive frameworks do not assume uniform needs across populations but promote flexibility, recognizing diverse conceptions of autonomy, care, and responsibility. In this way, regulation can enable safer digital participation while respecting the heterogeneity of users’ values and experiences.

#### 4.2.5 Antitrust and economic levers to reshape platform incentives

To shift the incentives driving aADs, economic and competition law tools are crucial. Pigouvian taxes on addictive features can internalize the social costs of excessive engagement and discourage exploitative design (Langvardt, 2019). While direct empirical evidence for such taxes specifically targeting older users is emerging, their robust economic theory of internalizing negative externalities, similar to successful precedents in tobacco or alcohol taxation, provides strong policy justification. Such taxes can serve as a pragmatic “second-best” policy, fostering gradual behavioral shifts and generating crucial real-world data for further optimization. Competition law could reframe aADs-caused psychological harms, like increased anxiety and digital dependency, as non-price consumer harms (Day, 2022). By recognizing the “attention market” as a relevant market for antitrust enforcement, regulators could treat bad-faith aADs practices as exclusionary practices. This expands the consumer welfare standard to include public health costs, redirecting corporate competition toward prioritizing user well-being over mere engagement maximization.

## 5 Conclusion

Regulating aADs must extend beyond youth-centric approaches to protect older adults. Without targeted safeguards, these technologies will continue to impact mental well-being, deepen the digital divide, and impose significant socio-economic costs. This paper draws primarily on Chinese case studies to offer a mirror through which to examine this issue, providing illustrative insights into emerging regulatory dilemmas rather than seeking to generalize from a single context. This article offers a normative analysis, calling for urgent policy attention to older adults. Future research should primarily focus on empirical analysis examining the relationships between older adults’ aADs engagement, their algorithmic awareness, the harms experienced, and the effectiveness of regulatory interventions. Advancing this agenda requires interdisciplinary collaboration among policymakers, technologists, public health experts, and social scientists. National regulators, such as digital regulatory bodies, health ministries, and consumer protection departments, should coordinate pilot programs and set research priorities. Multi-sector collaborations involving platforms, researchers, and civil society are essential. Funding could stem from public research grants, industry contributions, and international development funds focused on digital inclusion and fairness. Ensuring aADs do not undermine individual autonomy or public health should be a shared policy priority across aging digital societies.

## Data Availability

The original contributions presented in the study are included in the article/supplementary material, further inquiries can be directed to the corresponding author.
